# Artesunate monotherapy versus artesunate plus quinine combination therapy for treatment of imported severe malaria: a TropNet retrospective cohort study

**DOI:** 10.1007/s15010-022-01771-5

**Published:** 2022-02-27

**Authors:** Annarita Botta, Agnese Comelli, Iacopo Vellere, Flavia Chechi, Leila Bianchi, Gardini Giulia, Lina Rachele Tomasoni, Michele Spinicci, Luisa Galli, Francesco Castelli, Alessandro Bartoloni, Lorenzo Zammarchi

**Affiliations:** 1 Department of Experimental and Clinical Medicine, University of Florence, Florence, Italy; 2 University Department of Infectious and Tropical Diseases, University of Brescia and ASST Spedali Civili, Brescia, Italy; 3 School of Human Health Sciences, Degree of Medicine and Surgery, University of Florence, Florence, Italy; 4 Division of Pediatric Infectious Disease, Anna Meyer Children’s University Hospital, Florence, Italy; 5 Infectious and Tropical Diseases Unit, University Hospital Careggi, Florence, Italy; 6 UNESCO Chairholder “Training and Empowering Human Resources for Health Development in Resource-Limited Countries”, University of Brescia, Brescia, Italy; 7 ESCMID Study Group for Infections in Travellers and Migrants (ESGITM), Florence, Italy

**Keywords:** Malaria, Artemisinin resistance, Artesunate, Quinine, Italy

## Abstract

**Background:**

The addition of intravenous quinine (IVQ) to intravenous artesunate (IVA) has been recently suggested by World Health Organization  in areas where artemisinin resistance is highly prevalent. Since IVA is not yet widely available as “Good Manufacturing Practices” product, for several years combination treatment with IVA and IVQ was used in some Italian centers to mitigate the legal risks in using an unlicensed drug.

**Methods:**

A retrospective cohort study was designed to compare IVA + IVQ and IVA treatment for imported severe malaria. We collected data from three Italian centers. Adult and pediatric cohorts were analyzed separately.

**Results:**

Forty-nine patients treated with IVA and 44 with IVA + IVQ were enrolled, 45 were adults and 48 children. All acquired malaria in Sub-Saharan Africa. In the adult cohort, median of fever clearance time (FCT) was similar in both groups (48 h vs 48 h, *p* = 0.19) but number of patients who reached apyrexia within 48 h (FCT48) was higher in IVA group (20/24, 83.3% vs 8/17, 47%, *p* = 0.002). The parasite clearance time (PCT) measure did not differ (median 48 h vs 48 h, *p* = 0.669). In the pediatric cohort, FCT did not differ in the two groups (median 30 vs 48 h, *p* = 0.50) while PCT was longer in IVA + IVQ group (median 72 vs 48 h, *p* = 0.002). Adverse events (AEs) in adults were more common in the combination treatment group (6/19, 31.58% vs 2/26, 7.69%, *p* = 0.055).

**Conclusion:**

IVA + IVQ treatment did not show better outcome with respect to IVA monotherapy. AEs were more frequent in the IVA + IVQ group compared to the monotherapy. Further studies are necessary to investigate whether IVA + IVQ could be an efficient strategy to treat severe malaria cases in areas at high risk of artemisinin resistance.

**Supplementary Information:**

The online version contains supplementary material available at 10.1007/s15010-022-01771-5.

## Introduction

Malaria represents one of the most frequent cause of febrile illness in travelers returning from the tropics with about 8000 cases per year in Europe, and a case fatality rate of 0.8% [1]⁠. Since 2006, the World Health Organization (WHO) recommends intravenous artesunate (IVA) as treatment of choice for this condition since it has proven to be superior to intravenous quinine (IVQ) in reducing mortality in both adults and children in two well-known large randomized controlled trials performed in endemic countries [2, 3]⁠. However, the currently available IVA supplies are not being produced in accordance with the Good Manufacturing Practice (GMP) required by European Medicine Agency (EMA); therefore, the drug has not been licensed in Europe at this time and has to be imported from China or India. Despite the 2006 WHO recommendations, some European health care providers refused the use of a non-GMP product, especially in the first years after the guidelines publication. To minimize legal issues and to guarantee the best treatment, in some centers, a combined treatment protocol for severe malaria with IVA and IVQ was adopted [4]⁠. Later, this approach has been progressively discontinued, in virtue of increasing evidence of effectiveness and safety of non-GMP IVA also in non-endemic countries [5].

Since 2008, parasites with reduced sensitivity to artemisinin derivatives became increasingly prevalent in South-East Asia (Cambodia, Thailand, Vietnam, Myanmar, and Laos) [6].

This phenomenon, which leads to a delay in the clearance of parasites from the bloodstream of individuals treated with artemisinin derivatives, is currently a serious threat and may hinder efforts to tackle the disease. A major concern is that these resistant parasites will spread through Sub-Saharan Africa, the continent most affected by malaria (approximately 94% of cases), as happened with previous generations of antimalarial treatments (chloroquine and folic acid antagonists) [7]⁠. Indeed, a recent study  carried out in Uganda in 2017–2019 reported in vivo artemisinin resistance in 5.8% of patients (14 of 240) with most (13) of *Plasmodium falciparum* strains bearing a mutation on the *kelch13* gene. The prevalence of parasites with *kelch13* mutations increased significantly, from 3.9% in 2015 to 19.8% in 2019 [8, 9]⁠.

More recently, the combination treatment with IVA and IVQ has been used in severe malaria patients along the Thai Myanmar border where the resistance of *P. falciparum* to artemisinin derivatives is widespread [10]⁠. According to these authors, empirical addition of IVQ to IVA could represent a precautionary measure in these cases. Moreover, the WHO in the latest guidelines for malaria published in July 2021 recommended that parenteral artesunate and quinine to be given together in full doses for the treatment of severe malaria in areas with established artemisinin resistance [11].

Therefore, while combination treatment with IVA and IVQ could become a last resource to treat some patients coming from areas at risk for artemisinin derivatives resistance, it would be useful to collect additional information on the use of these two drugs in combination. To date there is only one randomized comparative study between IVA alone and IVA combined with IVQ for parenteral severe malaria treatment, reporting data on 69 patients in Thailand [12] and few smaller case series [4, 13]. According to the only existing randomized comparative study, the combination treatment was not more effective than the monotherapy with IVA and a higher rate of side effects was observed in patients who received IVQ [12].

In this study, we compared outcomes and adverse effects of severe malaria in children and adult patients treated with IVA monotherapy or IVA + IVQ combination regimen, observed during a 10-year period (2010–2019) in three tertiary care hospitals: the Department of Infectious and Tropical Diseases of the Spedali Civili ASST of Brescia (referred to as SC-BS), the Department of Infectious and Tropical Diseases of University Hospital, Florence (referred to as AOUC-FI), and the Department of Infectious Diseases of Meyer Children University Hospital, Florence (referred to as AOUM-FI).

## Methods

### Study design, setting and inclusion criteria

A multicenter retrospective comparative cohort study between the two therapeutic approaches (IVA and IVQ combined vs IVA monotherapy) for treatment of severe malaria was designed.

The study was carried out in the infectious diseases departments of three tertiary care hospitals, namely SC-BS, AOUC-FI and AOUM-FI.

Patients admitted to the three hospitals in the period 2010–2019, with a diagnosis of severe malaria according to the WHO criteria 2010 and 2015, were included in the study. Switch from IVA + IVQ combination therapy to IVA monotherapy for the treatment of severe malaria occurred in 2016, 2017 and 2019 at SC-BS, AOUC-FI and AOUM-FI, respectively.

More in detail, patients were categorized in one of the following treatment groups:

1. IVA 2.4 mg/kg bodyweight, then 2.4 mg/kg 12 h later, then 2.4 mg/kg/day. Starting form 2015, according to the WHO guidelines, for patients weighted less than 20 kg, IVA 3 mg/kg bodyweight, then 3 mg/kg 12 h later, then 3 mg/kg/day
2. IVA as reported above plus IVQ 20 mg/kg bodyweight loading dose, then 10 mg/kg every 8 h as maintenance dose.

When patients were able to take oral tablets, the therapy was switched to fixed dose oral artemisinin combination treatment (ACT) for 3 days.

### Variables and definitions

A severe case of malaria was defined by a positive blood smear for the asexual forms of *P. falciparum* or other *Plasmodium* spp. and the presence of one or more severity criteria according to the WHO guidelines 2010 or 2015. Of note, the threshold to define hyperparasitemia increased from > 2% in low intensity transmission areas (such as in Italy) according to the 2010 criteria, to 10% in all setting according to the 2015 criteria. We defined pediatric patients if aged under or equal 16.

For every case epidemiological data regarding age, sex, weight, comorbidities evaluated by Charlson Morbidity Index (CMI), country where most probably the infection was acquired, type and duration of treatment, number of days of presentation from onset of symptoms, type and duration of hospitalization (ICU or non-ICU setting) were retrospectively collected in a database to be further analyzed. Clinical data regarding most relevant signs and symptoms and laboratory test results like baseline hemoglobin (Hb), parasitemia, LDH, ALT, procalcitonin and C-reactive protein (CRP), use of antibiotic therapy were collected in the same way. Clinical variables namely jaundice, acute kidney injury (AKI) and hyperlacticaemia, hyperparasitemia were defined according to the criteria of severe malaria present in 2010 or 2015 WHO guidelines, depending on the year of diagnosis.

As outcome variables, we analyzed clinical and parasitological measures. Our primary outcomes were the clinical outcome measures including mortality rate, fever clearance time (FCT, i.e., the time from first dose of IVA received until the temperature decreased and remained below 37.5 °C for at least 48 h), fever clearance within 48 h (FCT48, i.e., number of patients not feverish after 48 h from first dose of IVA received), admission to intensive care unit (ICU) and length of hospitalization (both in ICU and non-ICU settings), mechanical and non-invasive ventilation, erythro-cyto-apheresis and occurrence of adverse effects (AE) such as tinnitus, hearing loss, dizziness, hypoglycemia, arrhythmias, prolongation of QT, hemolysis including post artesunate delayed hemolysis (PADH), hypertransaminasemia.

Secondary outcomes were the parasitological measures; we analyzed the parasitemia clearance time (PCT, i.e., the time elapsed between the patient's first dose of IVA and the time of the first negative blood smear) and parasitemia clearance after 48 h (PCT48, i.e., number of patients with the first negative blood smear after 48 h from the first dose of IVA received). The study was reviewed by the research committee of TropNet (European Network for Tropical Medicine and Travel Health) and approved by the ethical committees of the three centers (AOUC-FI study code CEAVC 15950, approved on November 12, 2019; AOUM-FI study code MALARIA, approved on April 28, 2020; SC-BS study code 3930, approved on May 21, 2020).

### Statistical methods

To minimize differences between age groups and to guarantee a homogeneous analysis of the collected data, adult and pediatric cohorts were analyzed separately. Continuous variables were summarized as medians and interquartile range (IQRs) and were compared using Mann–Whitney *U* tests for two-group comparison. Categorical variables were expressed as frequencies and percentages and were analyzed using Chi-squared or Fisher’s exact tests, as appropriate. Measures of categorical outcomes with *p* value equal or inferior to 0.2 were considered potentially significant and were further analyzed in a multivariate regression analysis, where appropriate. In the multivariate regression analysis, a *p* value inferior to 0.05 was considered significant. Stata 16.1 was used for all analyses.

## Results

We retrospectively enrolled 93 eligible patients, 49 treated with IVA and 44 treated with IVA + IVQ.

Eleven patients were enrolled in AOUC-FI, 19 in AOUM-FI and 63 in SC-BS. All patients fully recovered.

### Adult cohort

In total, 45 adult patients (26 treated with IVA and 19 with IVA + IVQ) were analyzed. Patients were predominantly male (30/45, 66.7%), with a median age of 43 (IQR 34–50). A large majority of patients had parasitemia > 2% (39, 86.7%) as criteria for severe malaria, according to 2010 WHO classification. Clinical features of severe malaria were found in a minority of patients: 16 with jaundice (35.5%), 4 with haemoglobinuria (8.9%), 4 with abnormal bleeding (8.9%), 4 with prostration (8.9%), 1 with respiratory failure (2.2%), 1 with impaired consciousness (2.2%). All of them were likely to have acquired the infection in Sub-Saharan Africa, with Nigeria the most represented country (10, 22.2%). In most cases, visiting relatives and friends (VRF) was the travel reason (33, 73.3%). *P. falciparum* was the species involved in all cases. Baseline characteristics of the two groups are described in Table 1.

**Table 1:** Baseline characteristics of adult patients at hospital admission sorted by intravenous artesunate and intravenous artesunate and quinine combined group

	Data available	IVA	IVA + IVQ	*p* value
*N*		26	19	
Age, median (IQR)	45	46 (38–56)	36 (30–46)	0.006
Male, *n* (%)	45	18 (69.2)	12 (63.2)	0.670
Weight, median (IQR)	45	76 (69–83)	73 (63–83)	0.251
Center	45			
AOUC-FI, *n* (%)		0	10 (52.6)	
SC-BS, *n* (%)		26 (100)	9 (47.4)	
Ethnic group	45			1.000
African, *n* (%)		19 (73.1)	14 (73.7)	
Caucasic, *n* (%)		7 (26.9)	5 (26.3)	
CMI, median (IQR)	45	1 (0–2)	0	0.003
Chemoprophylaxis in the last 12 weeks	45			0.422
No, *n* (%)		26 (100)	18 (94.7)	
Complete, *n* (%)		0	0	
Incomplete, *n* (%)		0	1 (5.3)	
Living in Africa in last 5 year	34	26	8	
*n* (%)		2 (7.7)	1 (12.5)	1.000
Severe malaria according to WHO 2010, *n* (%)	45	26 (100)	19(100)	
Severe malaria according to WHO 2015, *n* (%)	45	23 (88.5)	9 (47.4)	
Diagnosis				
*P. falciparum,* *n* (%)	45	26 (100)	19 (100)	
LDH, U/L baseline	35	20	15	
Median (IQR)		498 (365–721)	455 (279–570)	0.214
Hb, mg/dL baseline, median (IQR)	45	12.5 (11.3–14.0)	11.6 (10–14.1)	0.52
Parasitaemia, % baseline, median (IQR)	45	5.5 (3.9–8.5)	6 (3–20)	0.669
Hyperparasitaemia > 10%, *n* (%)	45	3 (11.54)	5 (26.32)	0.253
QTc, ms baseline	40	22	18	
Median (IQR)		423 (407.5–433.5)	415 (403–436)	0.754
Symptoms from returning to Italy	42	25	17	0.242
Before, *n* (%)		4 (15.4)	2 (10.5)	
Same day, *n* (%)		7 (26.9)	1 (5.3)	
1–3 days, *n* (%)		4 (15.4)	6 (31.6)	
4–7 days, *n* (%)		7 (26.9)	4 (21.1)	
8 or more day, *n* (%)		3 (11.5)	4 (21.1)	
Days from symptoms onset, median (IQR)	45	4 (3–6)	4 (3–6)	0.888
AKI, *n* (%)	45	4 (15.38)	4 (21.1)	0.704
Jaundice, *n* (%)	45	12 (46.2)	4 (21.1)	0.118
Patients with fever on admission, *n* (%)	45	24 (92.3)	17 (89.5)	0.741
Antibiotic therapy, *n* (%)	45	5 (19.2)	9 (47.4)	0.057

*IVA* intravenous artesunate, *IVQ* intravenous quinine, *CMI* Charlson morbidity index, *LDH* Lactate dehydrogenase, *AKI* acute kidney injury, *IQR* interquartile range

The two groups did not show any difference regarding sex, weight, use of chemoprophylaxis in last 12 weeks, laboratory values (LDH, Hb, parasitaemia on admission), days from symptoms onset and fever on admission. Regarding epidemiologic features, the IVA group had a significantly higher CMI (median 1 vs 0, *p* = 0.003) and was older (median 46 vs 36 years old, *p* < 0.006). Regarding clinical features, jaundice was more frequent in the IVA group (46.2% vs 21.1%, *p* = 0.118), whereas AKI and fever on admission were equally distributed.

Patients in the IVA + IVQ group received antibiotics more frequently (47.4% vs 19.2%, *p* = 0.057).

**Fig. 1:**
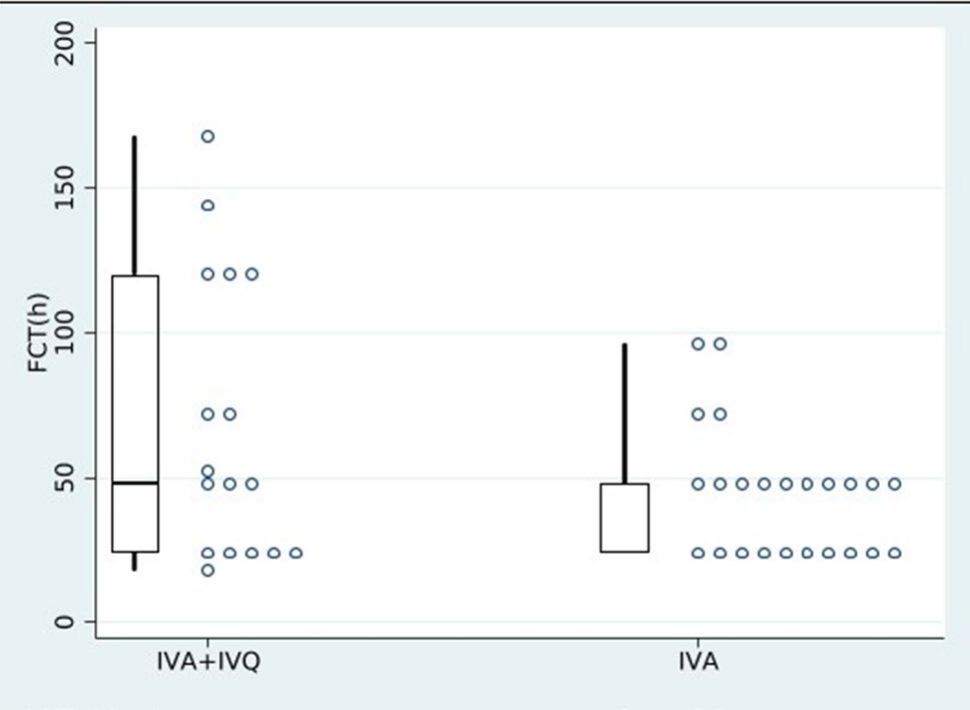
Box plot of fever clearance time (FCT) comparing intravenous artesunate (IVA) versus intravenous artesunate plus intravenous quinine (IVA + IVQ) groups in adult cohort

Clinical and parasitological outcomes are listed in Table 2. The median FCT was similar in both groups (median 48 h, *p* = 0.19) but number of patients who reached apyrexia within 48 h (FCT48) was significantly higher in IVA group (83.3%, *p* = 0.04) as shown in Fig. 1. This finding was confirmed in the multivariate analysis (Supplementary Table 1). Eight patients (17.8%) presented with AEs. Most of them were probably due to IVQ (6, 75%): they were all early-onset AE and discontinuation was necessary in all cases; there were no cases of hypoglycemia. IVA was deemed responsible for PADH in two patients (25%), a median 13 days after administration: one of them required blood transfusion (Table 3). Adverse events were more common in the combination treatment group (31.6 vs 7.7%, *p* = 0.055) but this finding was not confirmed in the multivariate analysis (Supplementary Table 2). PCT did not differ between the two groups (median 48 h in both groups, *p* = 0.669). No difference in length of hospitalization was observed (median 6 days, *p* = 0.402).

**Table 2:** Clinical and parasitological outcome following treatment of malaria adult patients sorted by intravenous artesunate and intravenous artesunate and quinine combined group

	Data available	IVA	IVA + IVQ	*p* value
*N*		26	19	
Clinical outcomes				
FCT	41	24	17	
Median (IQR)		48 (24–48)	48 (24–120)	0.19
FCT 48	41	24	17	
* n* (%)		20 (83.3)	8 (47)	0.002
Length of hospitalization, days median, (IQR)	45	6 (5–8)	6 (6–9)	0.402
Mechanic ventilation, *n* (%)	45	1 (3.8)	0	1.000
Non-invasive ventilation, *n* (%)	45	1 (3.8)	2 (10.5)	0.565
Erythrocytapheresis, *n* (%)	45	0	1 (5.3)	0.422
Admission to ICU, *n* (%)	45	3 (11.5)	1 (5.3)	0.432
Length of hospitalization in ICU setting, days, median (IQR)	45	1 (1–5)	4 (4–4)	0.637
AEs, *n* (%)	45	2 (7.69)	6 (31.58)	0.055
Parasitological outcomes				
PCT, median (IQR)	45	48 (48–48)	48 (48–72)	0.669
PCT 48, *n* (%)	45	20 (77)	13 (68)	0.717

*IVA* intravenous artesunate, *IVQ* intravenous quinine, *FCT* time to fever clearance in hours, *FCT48* number of patients not feverish after 48 h from first dose of IVA received, *ICU* intensive care unit, *AEs* adverse events, *PCT* time to parasite clearance in hours, *PCT48* number of patients with the first negative blood smear after 48 h from the first dose of IVA received

**Table 3:** Adverse effects in adults

Group	Description	Time to onset (days)	Duration (days)	AE toxicity grade	Countermeasure	Probable cause
IVA + IVQ	Hypoacusia	1	3	1	Discontinuation	Quinine
IVA + IVQ	Cinchonism	1	1	2	Discontinuation	Quinine
IVA + IVQ	QT elongation	0	1	2	Discontinuation	Quinine
IVA + IVQ	Cinchonism	1	1	2	Discontinuation	Quinine
IVA + IVQ	Cinchonism	1	1	1	Discontinuation	Quinine
IVA	Delayed hemolysis	10	8	2	Blood transfusion	Artesunate
IVA	Delayed hemolysis	16	9	1	Monitoring	Artesunate
IVA + IVQ	Cinchonism	1	3	2	Discontinuation	Quinine

*IVA* intravenous artesunate, *IVQ* intravenous quinine, *AE* adverse effect

### Pediatric cohort

In total, 48 cases (23 treated with IVA and 25 with IVA + IVQ combined therapy) were analyzed. All cases were classified as severe malaria according to WHO 2010 criteria: most of them presented parasitemia > 2% (42, 87.5%). A smaller part of patients presented clinical features typical of severe malaria: prostration in 9 (18.8%), coma was described in 4 patients (8.3%), jaundice in 3 (6.3%), circulatory collapse in 1 (2.1%). All infections were acquired in Sub-Saharan Africa, being Burkina Faso the most represented country (12, 25%). All patients were VRF. *P. falciparum* was the species involved in all patients: in particular, two patients (4.2%) presented mixed infection with *P. falciparum* and *P. vivax*. Baseline characteristics of the two groups are described in Table 4. As for epidemiological features, the two groups appeared homogeneous: they didn’t show differences for age (median 6 in both groups), gender (males were 56.5% in IVA group vs 64% in IVA + IVQ), and comorbidities. Regarding laboratory values, the two groups didn’t show significant differences: baseline parasitemia was similar in both groups (median 5% in IVA vs 4% in IVA + IVQ). Clinical features (fever at admission, jaundice, AKI) were similar in both groups. There was a not significant difference on the QTc at baseline (median 426 in IVA group vs 391.5 in IVA + IVQ, *p* = 0.185). Antibiotic therapy was administered to 76% of cases in IVA + IVQ group vs 17.4% in the IVA monotherapy group (*p* < 0.001).

**Table 4:** Baseline characteristics of pediatric cases at hospital admission sorted by treatment group

	Data available	IVA	IVA + IVQ	*p* value
*N*		23	25	
Age, median (IQR)	48	6 (3–9)	6 (2.5–9)	0.754
Male, *n* (%)	48	13 (56.5)	16 (64)	0.769
Weight, kg, median (IQR)	48	22 (14–47)	19 (15–31)	0.415
Center	48			
AOUC-FI, *n* (%)		0	1 (4.0)	
AOUM-FI, *n* (%)		0	19 (76)	
SC-BS, *n* (%)		23 (100)	5 (20)	
Ethnic group	48			
African, *n* (%)		23 (100)	25 (100)	
CMI, median (IQR)	48	0	0	
Prophylaxis in last 12 weeks	48			0.703
No, *n* (%)		16 (69.6)	20 (80)	
Complete, *n* (%)		3 (13)	2 (8)	
Incomplete, *n* (%)		4 (17.4)	3 (12)	
Living in Africa in last 5 year	28	23	5	
* n* (%)		4 (17.4)	2 (40)	0.285
Severe malaria WHO 2010, *n* (%)	48	23 (100)	25 (100)	
Severe malaria WHO 2015, *n* (%)	48	20 (87)	6 (24)	
Diagnosis	48			
* P. falciparum,* *n* (%)		22 (95.7)	24 (96)	
* P. falciparum* + *P. vivax*, *n* (%)		1 (4.3)	1(4)	
LDH, U/L baseline	36	20	16	
Median (IQR)		409.5 (351.3–526.8)	502 (354.3–654.8)	0.484
Hb, mg/dL baseline, median (IQR)	48	10.4 (7.8–12.1)	9.1 (8–11.1)	0.502
Parasitemia, % baseline, median (IQR)	48	5 (2.2–9)	4 (2.5–8)	0.456
Hyperparasitemia > 10%	48	23	25	
* n* (%)		4 (17.39)	3 (12)	0.696
QTc, ms baseline	31	13	18	
Median (IQR)		426 (411–433.5)	391.5 (361–429.8)	0.185
Symptoms from returning to Italy	48			0.394
Before, *n* (%)		0	6 (24)	
Same day, *n* (%)		4 (17.4)	2 (8)	
1–3 days, *n* (%)		5 (21.7)	3 (12)	
4–7 days, *n* (%)		4 (17.4)	5 (20)	
8 or more days, *n* (%)		9 (39.1)	9 (36)	
Days from symptoms onset, median (IQR)	48	3 (2–5)	4 (2.5–7)	0.277
AKI, *n* (%)	48	1 (4.3)	1 (4)	1.000
Hyperlactacidemia, *n* (%)	48	0	3 (12)	0.235
Jaundice, *n* (%)	48	3 (13)	2 (8)	0.660
Patients with fever on admission, *n* (%)	48	17 (73.9)	20 (80)	0.616
Antibiotic therapy, *n* (%)	48	4 (17.4)	19 (76)	< 0.001

*IVA* intravenous artesunate, *IVQ* intravenous quinine, *CMI* Charlson morbidity index, *AKI* acute kidney injury

**Fig. 2:**
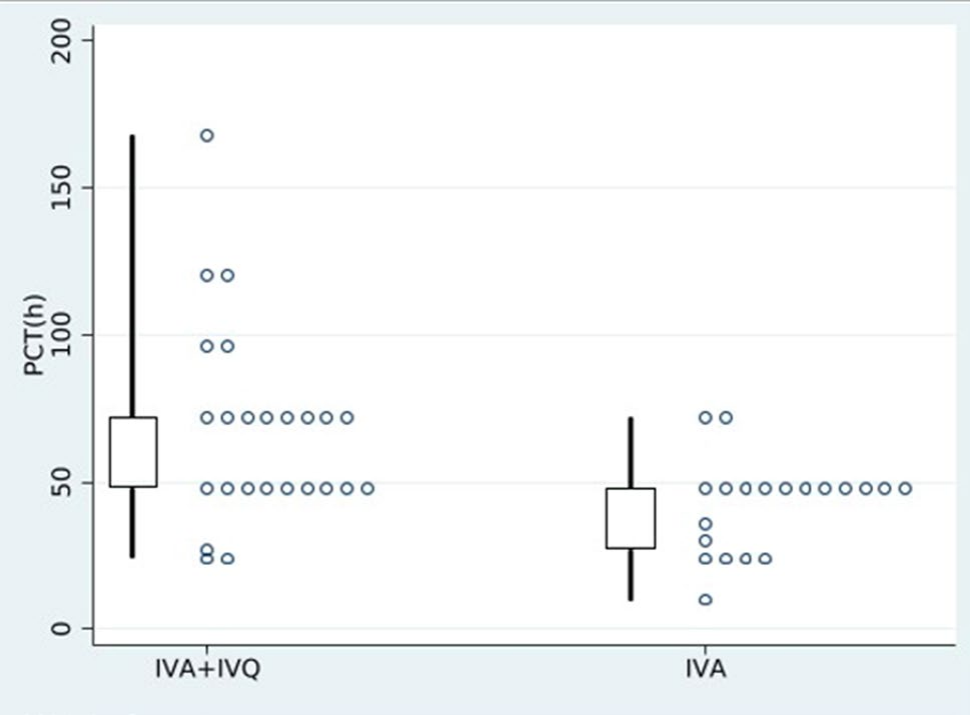
Box plot of parasite clearance time (PCT) comparing intravenous artesunate (IVA) versus intravenous artesunate plus intravenous quinine (IVA + IVQ) groups in pediatric cohort

Clinical and parasitological outcomes are listed in Table 5*.* PCT and admission to ICU differed between the two groups. PCT was higher in IVA + IVQ group (median 72 vs 48 h, *p* = 0.002). Likewise, PCT48 was lower in IVA + IVQ group, in comparison with IVA group (48% vs 90%, respectively, *p* < 0.004) (Fig. 2). Sixteen (69.6%) patients were admitted to ICU in IVA group vs 4 (16%) in IVA + IVQ group (*p* = 0.023). FCT and AEs were not significantly different between the two groups. The median of FCT was 30 h in IVA group *vs* 48 in IVA + IVQ group (*p* = 0.5). The AEs reported were represented by 2 cases of PADH, one in each group, with an average time of onset of 12 days. The watch and wait management was the strategy adopted in both cases. No difference in length of hospitalization was observed (*p* = 0.505).

**Table 5:** Clinical and parasitological outcome measures of pediatric cases sorted by treatment group

	Data available	IVA	IVA + IVQ	*p* value
*N*		23	25	
Clinical outcomes				
FCT	47	17	20	
Median (IQR)		30 (24–96)	48 (24–84)	0.50
FCT 48	47	17	20	
*n* (%)		12 (70.59)	13 (65)	1
Length of hospitalization, days median (IQR)	48	6 (5–6)	6 (5–7)	0.505
Mechanic ventilation, *n* (%)	48	0	0	
Non-invasive ventilation, *n* (%)	48	0	2 (8)	0.49
Erythrocytapheresis, *n* (%)	48	0	6 (24)	0.023
Admission to ICU, *n* (%)	48	16 (69.6)	4 (16)	< 0.001
Length of hospitalization in ICU setting, days, median (IQR)	48	1 (1–2)	1.5 (1–3.5)	0.774
AEs, *n* (%)	48	1 (4.35)	1 (4)	1
Parasitological outcomes				
PCT	45	20	25	
Median (IQR)		48 (27–48)	72 (48–72)	0.002
PCT 48	45	20	25	
* n* (%)		18 (90)	12 (48)	0.004

*IVA* intravenous artesunate, *IVQ* intravenous quinine, *FCT* time to fever clearance in hours, *FCT48* number of patients not feverish after 48 h from first dose of IVA received, *ICU* intensive care unit, *AEs* adverse events, *PCT* time to parasite clearance (hours), *PCT48* number of patients with the first negative blood smear after 48 h from the first dose of IVA received

## Discussion

The introduction of artemisinin and its derivatives represented a cornerstone in the therapeutic approaches against malaria, resulting the more effective antimalarial agents. A Cochrane meta-analysis suggested that IVA should replace IVQ as treatment of choice for severe malaria worldwide [14]⁠. Indeed, IVA allowed a faster clearance of malaria parasites than other agents, and reduced gametocyte carriage, thus decreasing the potential of malaria transmission [15]⁠. However, the effect on mortality has never been formally assessed in non-endemic setting, where randomized comparative clinical trials are no more possible for ethical issues. European observational studies demonstrated a clear effect on parasitemia clearance time and length of stay in ICU [16]⁠. In the United States the mortality observed in a cohort study of patient with severe malaria treated with IVA was lower (1.8%) than for patients with severe malaria treated with quinidine (3.0%) before the use of IVA implemented in this country [17]. At the same time, it is well known the emergence of resistance to artesunate in South-East Asia, and warnings about the possible emergence of malaria parasites capable of resisting artemisinin derivatives in Sub-Saharan Africa are increasing over time. In such evolving scenario, IVQ may still have a role in both endemic and non-endemic settings, as recently observed [18], including its use as part of a combination treatment with IVA. The WHO malaria guidelines published in July 2021 recommend that parenteral artesunate and parenteral quinine to be given together in full doses for the treatment of severe malaria in areas with established artemisinin resistance [11]. Recently a randomized clinical trial evaluating the efficacy and safety of dual intravenous artesunate plus quinine compared to intravenous artesunate for cerebral malaria in Nigerian children (DUAL PAQ TRIAL Protocol) has been designed underling the interest on additional data on combination treatment in certain clinical settings [19].

In this recent perspective, we compared, retrospectively, IVA monotherapy and IVA + IVQ combination therapy for severe imported malaria in both adult and pediatric subjects. All patients reached full recovery. In the adult subgroup we found that patients receiving the combination therapy took a longer time to clear fever, while no difference in time to parasite clearance was observed between the two groups. These data may suggest that there is no additive effect of quinine, notably slower to act, to the parasiticidal activity of IVA, which allows a faster in vivo parasite clearance, and a more rapid antipyretic effect. The correlation between parasitemia levels and number of patients who reach fever clearance time at 48 h, comparing the two groups was more evident for intermediate level of parasitemia (5–9%) compared with lower (< 5%) or higher levels (≥ 10%) (Supplementary Table 3).

Unfavorable pharmacokinetic or pharmacodynamic interaction between the two antimalarial drugs cannot be excluded: IVQ undergoes an extensive hepatic biotransformation by CYP450 family (in particular, via CYP3A4, CYP2C9 and others), while IVA is hydrolyzed to dihydroartemisinin (DHA) by plasma esterase with a possible contribution of CYP2A6 enzymes, and then cleared by glucuronidation. The use of the CYP pathway of both drugs may be a possible explanation for the absence of a synergistic effect and the slowdown of IVA activity, due to the saturation of hepatic substrates by IVQ: it is known that IVQ is a weak inhibitor of CYP2A6, although this could be balanced with its inhibitory effect on glucuronidation [20, 21]. On the other hand, DHA induces CYP3A4, which is the main responsible for IVQ clearance [22]. The mentioned interactions could explain the lower performance of IVA when combined with IVQ.

The larger use of antibiotic therapy in the combination group may reflect the different prescriptive habit of the centers since the sub-analysis reveals that the majority of antibiotics have been prescribed by centers (AOUC-FI and AOUM-FI) which more frequently used the combination therapy. Adverse effects were more common in IVA + IVQ group, as expected, without reaching a level of statistical significance. Comparative studies from literature showed a higher incidence of hypoglycemia and QT elongation in patients treated with IVQ with respect to those receiving IVA [13, 16, 23]. In our cohort, no case of hypoglycemia was found, while we imputed the only case of QT elongation’s to quinine because it leads to changes in cardiac electrophysiology [23]. A comparative analysis showed that while IVQ can cause several different adverse effects, PADH was present only in patients treated with IVA, alone or in combination with IVQ, thus requiring an appropriate follow-up [23]⁠. Likewise, we observed two PADH in the IVA group, one of which required blood transfusion. PADH has been shown to be linked to the “pitting” phenomenon, i.e., the splenic removal of dead parasites from their host erythrocytes. The proportion of pitted erythrocytes is higher after the use of artemisinin than quinine [23]. We may hypothesize that IVQ may induce some protection on pitted erythrocytes by reducing their extraction during splenic passage. This phenomenon could help to explain the incidence only in the IVA group.

In the only comparative studies between IVA and IVQ vs IVA monotherapy, a significant difference in terms of AEs was found, but it is not clear whether or not patients were followed-up [12].

In pediatric population, clinical and parasitological outcomes did not differ between the two groups. In particular, FCT was similar (median 30 h in IVA group vs 48 h in IVA + IVQ). PCT was different in the two groups (median 48 h in IVA group vs 72 h in IVA + IVQ) and PCT48 was lower in the combination group (48% vs 90%) confirming the possible interaction between the two drug in the combination strategy which leads to a slower clearance of parasitemia. To our knowledge, there are no comparative studies between IVA and IVA + IVQ in a pediatric population. In our pediatric cohort the 2 cases of PADH (4.1%) detected were self-limiting and occurred in patients with high parasitemia (12% and 8%, respectively). These data are consistent with literature that shows a positive relationship between parasitemia and PADH [23, 24]. Indeed, according to two previous reports on children with severe malaria treated in a non-endemic country, PADH was reported in 1/3 and 3/9 of cases, respectively, with higher incidence of PADH occurring in patients with a high level of parasitemia [25, 26].

Our study presents several limitations. The main limitations of this study are small number of cases included, and the retrospective design with its intrinsic selection and recall bias. Other limitations deserve to be mentioned: (a) patients were managed in the three different centers; (b) the small sample size may explain why AEs were more common in the IVA + IVQ group but in the multivariate analysis did not reach a level of significancy; (c) the coadministration of antibiotic therapy was more common in the IVA + IVQ group and this may lead to more AEs. Moreover, only subjects coming from Africa were included, an area with a low risk of artemisinin resistance, so we cannot exclude an advantage of combination treatment in patients who have acquired the infection in areas where artemisinin resistance is widespread. The application of different protocols depending on the centers, could be an additional limitation; SC-BS, for example, has a more aggressive approach sending preventively severe malaria cases to ICU for at least one night. Of note, to the best of our knowledge this is the first study which compare combination treatment in a pediatric cohort.

## Conclusions

In conclusion, our data are consistent with a RCT which compared the IVA alone with IVA plus IVQ: the combination treatment did not show a better outcome compared with IVA alone. IVA + IVQ did not accelerate parasite clearance and was not beneficial from the clinical point of view. Moreover, AEs were more frequent in the IVA + IVQ group compared to the monotherapy. Further larger and possibly randomized studies are necessary to investigate if the combination of IVA + IVQ could be an efficient empirical strategy to treat severe malaria cases acquired in areas where artemisinin resistance is widespread.

## Supplementary Information

Below is the link to the electronic supplementary material.

Supplementary file1 (DOCX 21 kb)
