# Supplementary material for: Artesunate monotherapy versus artesunate plus quinine combination therapy for treatment of imported severe malaria: a TropNet retrospective cohort study
Source: Infection. 2022 Feb 27;50(4):949–58. doi: 10.1007/s15010-022-01771-5 (PMC9338132; doi:10.1007/s15010-022-01771-5)
Supplement: Supplementary file 1 — Supplementary file1 (DOCX 21 kb) [file 15010_2022_1771_MOESM1_ESM.docx]

**Supplementary material**

*Supplementary Table 1. Logistic regression analysis on FCT at 48 hours in adult cohort*

| FCT 48 | aOR | 95% CI | **p value** |  |
| --- | --- | --- | --- | --- |
| IVA | 10.58 | 1.07-104.87 | 0.04 |  |
| Age | 0.99 | 0.89-1.10 | 0.823 |  |
| CMI | 3.13 | 0.45-21.91 | 0.250 |  |
| Jaundice | 0.13 | 0.01-1.22 | 0.07 |  |

Footnotes: FCT48 = number of patients not feverish after 48 hours from first dose of IVA received; IVA=intravenous artesunate; CMI=Charlson morbidity index

*Supplementary Table 2. Logistic regression analysis on AE in adult cohort*

| AEs | aOR | 95% CI | **p value** |  |
| --- | --- | --- | --- | --- |
| IVA | 0.29 | 0.03-2.67 | 0.273 |  |
| Age | 1.02 | 0.92-1.14 | 0.697 |  |
| CMI | 0.64 | 0.13-2-93 | 0.561 |  |

Footnotes: AEs= adverse events; IVA=intravenous artesunate; CMI=Charlson morbidity index

*Supplementary Table 3. Correlation between parasitemia levels and fever clearance time at 48h in the adult cohort*

| **Parasitemia <5%** | FCT48 | **p value** |
| --- | --- | --- |
| IVA group (n,%) | 9(75) |  |
| IVA+IVQ group (n,%) | 4 (57.14) |  |
|  |  | P=0.617 |
| **Parasitemia 5-9%** |  |  |
| IVA group (n,%) | 7 (87.5) |  |
| IVA+IVQ group (n,%) | 1 (20) |  |
|  |  | P=0.032 |
|  |  |  |
| **Parasitemia>/=10%** |  |  |
| IVA group (n,%) | 4 (100) |  |
| IVA+IVQ group (n,%) | 3 (60) |  |
|  |  | P=0.444 |

Footnotes: IVA= intravenous artesunate ; FCT48 = number of patients not feverish after 48 hours from first dose of IVA received; IVQ= intravenous quinine
